# Fine map of the *Gct1* spontaneous ovarian granulosa cell tumor locus

**DOI:** 10.1007/s00335-012-9439-6

**Published:** 2012-11-18

**Authors:** Kerri N. Smith, Sarah J. Halfyard, Edward S. Yaskowiak, Kathryn L. Shultz, Wesley G. Beamer, Ann M. Dorward

**Affiliations:** 1 Division of BioMedical Sciences, Faculty of Medicine, Memorial University of Newfoundland, St. John’s, NL A1B 3V6 Canada; 2 The Jackson Laboratory, Bar Harbor, ME 04609 USA

## Abstract

The spontaneous development of juvenile-onset, ovarian granulosa cell (GC) tumors in the SWR/Bm (SWR) inbred mouse strain is a model for juvenile-type GC tumors that appear in infants and young girls. GC tumor susceptibility is supported by multiple *Granulosa cell tumor* (*Gct*) loci, but the *Gct1* locus on Chr 4 derived from SWR strain background is fundamental for GC tumor development and uniquely responsive to the androgenic precursor dehydroepiandrosterone (DHEA). To resolve the location of *Gct1* independently from other susceptibility loci, *Gct1* was isolated in a congenic strain that replaces the distal segment of Chr 4 in SWR mice with a 47 × 10^6^-bp genomic segment from the Castaneus/Ei (CAST) strain. SWR females homozygous for the CAST donor segment were confirmed to be resistant to DHEA- and testosterone-induced GC tumorigenesis, indicating successful exchange of CAST alleles (*Gct1* ^*CA*^) for SWR alleles (*Gct1* ^*SW*^) at this tumor susceptibility locus. A series of nested, overlapping, congenic sublines was created to fine-map *Gct1* based on GC tumor susceptibility under the influence of pubertal DHEA treatment. Twelve informative lines have resolved the *Gct1* locus to a 1.31 × 10^6^-bp interval on mouse Chr 4, a region orthologous to human Chr 1p36.22.

**Electronic supplementary material:**

The online version of this article (doi:10.1007/s00335-012-9439-6) contains supplementary material, which is available to authorized users.

## Introduction

Human granulosa cell (GC) tumors are sex cord-stromal ovarian tumors that are divided into two clinicopathologic subtypes, adult-type and juvenile-type, based historically on histology and age at tumor diagnosis. Adult-type GC tumors are the more common subtype and typically occur in peri- and postmenopausal women (Björkholm and Silfverswärd 1981). Juvenile-type GC tumors can appear in girls from the time of infancy through adolescence and present immediate endocrinological and reproductive complications in addition to malignant potential (Young et al. 1984). A significant advance in our understanding of the GC tumorigenic mechanism has come with the introduction of high-throughput sequencing technology and the identification of a somatic missense mutation (p.C134W) in the *FOXL2* gene that is common and specific for adult-type GC tumors (Shah et al. 2009; Al-Agha et al. 2011). The *FOXL2* somatic mutation is not strongly associated with the juvenile subtype, suggesting alternate pathways for tumorigenic initiation in the juvenile-onset cases. The development of spontaneous juvenile-onset GC tumors in females of the SWR/Bm (hereafter SWR) inbred mouse strain is a model genetic system to identify pathways leading to juvenile-onset GC tumorigenesis.

The SWR inbred mouse strain and related recombinant inbred (RI) strains are unique for their heritable and spontaneous GC tumor phenotype that affects 1–25 % of the young female population, depending on strain background and hormonal stimulation (Beamer et al. 1985). In affected females, GC tumors develop during a restricted initiation window that coincides with puberty (3–4 weeks of age) as preneoplastic blood-filled follicles that are macroscopically visible on the ovarian surface (Tennent et al. 1990). By 6–8 weeks of age, the tumors progress to highly vascularized cystic or hemorrhagic masses that are homogeneously composed of GCs and enclosed within the ovarian bursa (Beamer et al. 1985; Tennent et al. 1990). Those females that progress through the pubertal transition unaffected remain tumor-free, retain fertility with average litter sizes, and age normally without significant predisposition for the development of other primary tumors. Those females with initially benign proliferative tumors have a high risk for development of malignant disease, with a time course for progression between 6 and 10 months of age (Beamer et al. 1985; Tennent et al. 1990).

The mouse GC tumors are endocrinologically active, secreting high levels of estrogen and inhibin (Beamer et al. 1988a; Gocze et al. 1997), similar to their human juvenile GC tumor counterparts (Young et al. 1984; Sivasankaran et al. 2009). Spontaneous GC tumor initiation in SWR mice is also endocrine-sensitive, as demonstrated by tumor grafting studies that have determined that an intact hypothalamic-pituitary-gonadal axis is required for GC tumor development (Beamer et al. 1993). In the intact SWR female mouse, tumor frequency is significantly increased from <1 to ≥20 % with exogenous androgen supplementation such as dehydroepiandrosterone (DHEA) and testosterone (Beamer et al. 1988a, b; Tennent et al. 1993; Dorward et al. 2007). In accordance with the lack of spontaneous GC tumor development outside the restricted susceptibility window, postpubertal treatment with these hormones does not stimulate GC tumor initiation (Beamer et al. 1993). Furthermore, short-term exposure to 17β-estradiol before, but not after, the appearance of preneoplastic follicular lesions suppresses GC tumor incidence (Dorward et al. 2007), indicating that the window of tumor initiation overlaps with that of tumor prevention in the mouse model. The restricted pubertal window also suggests that the first wave of maturing follicles have a distinct response to endocrine stimulation relative to subsequent waves of maturing follicles. A recent report by Mork et al. (2012) describes temporal differences in GC specification in the embryonic ovary, which may contribute to phenotypic differences and developmental sensitivity of the ovarian follicle responses in SWR tumor-susceptible females at puberty.

Three previous mapping studies designed to identify genes associated with GC tumor development in SWR mice determined that tumor susceptibility is a polygenic trait involving multiple GC tumor (*Gct*) susceptibility loci. The first mapping strategy examined the tumor susceptibility traits of 14 lines of the SWXJ RI strain set, which carry different homozygous chromosomal combinations of the progenitor SWR and SJL/Bm (hereafter SJL) inbred strains (Svenson et al. 1995; Shultz et al. 1996). Female mice that retained spontaneous or androgen-induced GC tumor susceptibility always retained the SWR-derived *Gct1* locus on Chr 4 (Beamer et al. 1988b; Tennent et al. 1993). It was also determined from the SWXJ strain set that the influence of DHEA at *Gct1* is genetically distinguishable from the action of testosterone at *Gct4* on Chr X, suggesting that *Gct1* is a discrete target for DHEA in female mice that carry the *Gct1* ^*SW*^ alleles (Beamer et al. 1988b). An F_2_ intercross mapping study using the SWXJ RI parental strains SWR and SJL confirmed significant linkage of GC tumor susceptibility with SWR alleles at *Gct1* on Chr 4, along with *Gct2* on Chr 12, *Gct3* on Chr 15, and *Gct5* on Chr 9, and confirmed the modifier influence of SJL alleles at *Gct4* on Chr X (Beamer et al. 1998). To improve genetic resolution, an N_2_F_1_ backcross mapping strategy between the GC tumor–susceptible SWXJ-9 RI strain and the evolutionarily divergent *Mus musculus castaneus* (CAST) strain confirmed *Gct1* as the fundamental locus for tumor initiation contributed by SWR and identified three novel autosomal loci, *Gct7* on Chr 1, *Gct8* on Chr 2, and *Gct9* on Chr 13, and two epistatic interactions between loci on Chrs 17 and 18 and between loci on Chrs 2 and 10 (Dorward et al. 2005). In this backcross, tumor susceptibility alleles were consistently contributed by the Swiss-derived strains (SWR or SJL) that contribute to the SWXJ-9 genome, with no GC tumor susceptibility alleles contributed by the CAST strain.

The required contribution of SWR-derived susceptibility alleles at *Gct1* (*Gct1* ^*SW*^) to support both spontaneous and androgen-induced GC tumorigenesis across strain backgrounds underscored the *Gct1* ^*SW*^ allele as an essential driver for this complex and developmentally restricted ovarian tumor phenotype. To map *Gct1* independently from the other autosomal loci, we constructed a congenic mouse strain that has a SWR genomic complement with the exception of a homozygous Chr 4 segment derived from the CAST strain in the region of *Gct1*. The congenic line was resistant to GC tumor development under androgenic influence, and therefore it was useful to support a subcongenic mapping strategy to refine the interval harboring *Gct1*. In this report, we present a high-resolution genetic map for the *Gct1* ovarian tumor susceptibility locus and a list of candidate genetic determinants for early-onset GC tumorigenesis.

## Materials and methods

### Mouse housing and nutrition

Mice were maintained in the specific-pathogen-free barrier facility in the Faculty of Medicine at Memorial University of Newfoundland and housed under a 12:12-h light/dark cycle. All mice were provided Laboratory Autoclavable Rodent Diet 5010 food (27.5 % protein, 13.5 % fat, 59 % carbohydrate; PMI Nutrition International, Richmond, IN, USA) and autoclaved water *ad libitum*. Females were weaned at 20–23 days of age and housed in groups of two to five animals per cage, which were 27.9 cm (L) × 17.8 cm (W) × 12.7 cm (H) with high-profile filtered lids and contained sterilized Bed-O-Cobs^®^ corn-cob bedding material (The Andersons, Maumee, OH, USA). All animal procedures were approved by the Institutional Animal Care Committee of Memorial University of Newfoundland.

### SWR.SJL-X.CAST-4 congenic strain development

SWR.SJL-X_5_ is a previously reported congenic strain that carries the *Gct1* ^*SW*^ alleles on Chr 4 and *Gct4* alleles on Chr X derived from the SJL strain (Beamer et al. 1998; Dorward et al. 2003). SWR.SJL-X_5_ female mice exhibit increased penetrance for spontaneous GC tumor initiation (~20 %) with the same pubertal initiation timing as SWR females, without the requirement for exogenous androgen administration. A large segment of Chr 4 from the CAST strain was transferred onto the SWR.SJL-X_5_ congenic strain background through ten consecutive backcross generations followed by brother–sister intercross generations to create the unique strain SWR.SJL-X.CAST-4T (hereafter Line 4-T). The CAST homozygous donor segment of Line 4-T spans the region *D4Mit31*–*D4Mit256*, a 47.6 × 10^6^-bp interval that includes the confidence intervals previously determined for the *Gct1* locus (Beamer et al. 1998; Dorward et al. 2003). The polymorphic CAST strain was chosen as a donor strain in the congenic mapping scheme to facilitate high-resolution genetic mapping of *Gct1*. The N_10_ backcross scheme also replenished the SWR.SJL-X_5_ genomic background (estimated return of >99 % host genomic material). Established mating pairs of Line 4-T were confirmed to possess SWR allelic background at other mapped *Gct* susceptibility loci on Chrs 9, 12, and 15 and SJL allelic background at *Gct4*. Recombinant subcongenic lines were produced by mating Line 4-T females with SWR.SJL-X_5_ males to produce progeny heterozygous for CAST and SWR on Chr 4 between *D4Mit31* and *D4Mit256*. Subsequent backcrossing to SWR.SJL-X_5_ generated N_2_F_1_ mice with unique combinations of the CAST and SWR genomes across the *Gct1* locus via meiotic recombination. Selected recombinants were chosen as founders for nested and overlapping subcongenic lines that were fixed to homozygosity through brother–sister matings prior to phenotype assessment for GC tumor susceptibility.

### Phenotyping

Granulosa cell tumor initiation is a spontaneous, low-penetrance trait in female SWR mice that is sensitive to the androgenic environment (Beamer et al. 1985). Both DHEA and testosterone treatments increase trait penetrance in SWR females and GC tumor–susceptible SWXJ strains with the expectation for different genetic targets on Chr 4 and Chr X, respectively (Beamer et al. 1988b). To test the GC tumor initiation phenotype of Line 4-T females, DHEA or testosterone was administered to prepubertal females (age = 20–24 days) in the form of a subcutaneous capsule. DHEA (5-androsten-3β-ol-17-one) or testosterone (4-androsten-17β-ol-3-one) (Steraloids Inc., Newport, RI, USA) was packed into 1.0-cm capsules made from Silastic tubing (1.98-mm inner diameter × 3.18-mm outer diameter; Dow Corning, Midland, MI, USA) capped with glass beads, as previously described (Beamer et al. 1988a). Capsules were implanted subcutaneously on the back at the time of weaning under isoflurane anesthesia (Baxter Corporation, Mississauga, ON, Canada) with postoperative carprofen (5 mg/kg body weight [bw]) analgesic (Pfizer Canada, Kirkland, QC, Canada). For phenotypic examination, female mice were necropsied at 8 weeks of age and ovaries were examined for GC tumors. At this age, tumors present as unilateral or bilateral, cystic or solid hemorrhagic masses of 5–10 mm^3^ that are macroscopically identifiable. Females with either unilateral or bilateral GC tumors were counted as one affected animal. Following confirmation of the GC tumor-resistant phenotype in Line 4-T females, all subcongenic line females were administered the DHEA capsule treatment to categorize the lines as tumor-susceptible or tumor-resistant.

### Genotyping

Genomic DNA isolated from tail tip biopsy was genotyped with a series of 22 SSLP markers around *Gct1* using a Master*Taq* Kit (5 PRIME Inc., Gaithersburg, MD, USA). Each PCR reaction contained 7.3 μL of distilled water, 1 μL of 5 × *Taq*Master PCR Enhancer heated to 65 °C, 1 μL of 10 × Reaction Buffer, 0.2 μL of 10 mM dNTPs (Invitrogen, Carlsbad, CA, USA), 0.22 μL each of 10 μM forward and reverse primers (Integrated DNA Technologies, Coralville, IA, USA), 0.05 μL of *Taq* DNA polymerase, and 1 μL of DNA template. PCR conditions were as follows: 97 °C for 30 s; 39 cycles of 94 °C for 15 s, 55 °C for 30 s, and 72 °C for 30 s; 72 °C for 10 min. Informative markers were determined using the Mouse Genome Database (Eppig et al. 2012) and included *D4Mit31*, *D4Mit279*, *D4Mit70*, *D4Mit148*, *D4Mit179*, *D4Mit13*, *D4Mit126*, *D4Mit160*, *D4Mit232*, *D4Mit233*, *D4Mit206*, *D4Mit129*, *D4Mit49*, *D4Mit362*, *D4Mit127*, *D4Mit190*, *D4Mit208*, and *D4Mit256*. PCR-based DNA markers were also designed in-house around novel SSLPs: *D4sjh19* (5’-GTG GGG AGC ATG TCC TTA AA and 5’-TGG GAG TGT GTA GCA GAG GA), *D4sjh10* (5’-GCA GAA ATG GCA CAG GAG AT and 5’-CCC ACA TTT GAA ACC ACC TC), *D4sjh12* (5’-TTT GGA GAC CAA AGA TGA GAC A and 5’-CTT TTC TGG CCT CTG ACC TG), and *D4sjh13* (5’-TCT GGT TTC TAT GCG TGT GC and 5’-CGG GAT CCA TAT GGT AGT GG). SSLP PCR products were separated by horizontal electrophoresis in 4 % MetaPhor agarose slab gels (Lonza, Rockland, ME, USA). The bands were visualized by ethidium bromide staining and were photographed for allele scoring purposes.

To facilitate fine mapping of *Gct1*, additional genetic polymorphisms between the CAST and SWR genomes were determined to supplement annotated DNA markers. Primer sequences were designed around known single nucleotide polymorphisms (SNPs) annotated in dbSNP (Sherry et al. 2001), or made use of unique SNPs identified during preliminary candidate gene sequencing (Supplementary Table 1). Because the SWR strain sequence is not publicly available, annotated SNPs were chosen on the basis of differences between CAST alleles and the alleles of other common inbred strains, particularly Swiss-derived strains that are phylogenetically related to SWR (Petkov et al. 2004). Each PCR reaction contained 22.5 μL of Accuprime™ Pfx Supermix, 0.25 μL each of 10 μM forward and reverse primers, and 2 μL of salt-extracted kidney DNA template. PCR conditions were as follows: 95 °C for 5 min; 35 cycles of 95 °C for 15 s, 55 °C for 30 s, and 68 °C for 45 s; 72 °C for 10 min. The amplicons were sequenced in both directions using a BigDye Terminator v3.1 Cycle Sequencing Kit (Applied Biosystems, Foster City, CA, USA), on a 3730 DNA Analyzer and aligned with published sequences using Sequencher ver. 4.10.1 (Gene Codes Corporation, Ann Arbor, MI, USA).

### Statistical analysis

A sufficient sample size per subcongenic line was determined to be 50 individual females based on statistical power calculations using an estimated DHEA-induced GC tumor incidence of 20 % in susceptible lines relative to a tumor-resistant phenotype of 0 % (Beamer et al. 1988b). Type I and Type II error probabilities were set to *α* = 0.05 and *β* = 0.20, respectively. The GC tumor incidence of each subcongenic line was individually compared to the SWR inbred line incidence using the χ^2^ analysis for proportions, with a chosen significance level of *P* *<* 0.05. All statistical analyses were performed with GraphPad Prism ver. 5.00 (GraphPad Software, San Diego, CA, USA).

## Results

### GC tumor incidence in SWR.SJL-X.CAST-4 founder congenic and subcongenic lines

Following the construction of the homozygous congenic Line 4-T, female mice were tested for GC tumor susceptibility under the influence of testosterone or DHEA, as per previous studies with SWR inbred and SWXJ RI strain females. Neither DHEA nor testosterone administration in the form of subcutaneous capsule implants triggered GC tumor development in Line 4-T females (Table 1). In direct contrast, SWR females exhibited a GC tumor incidence of 18 % with DHEA capsule administration, in keeping with historical incidence following androgen supplementation in the diet (Table 1; Beamer et al. 1993). The GC tumor-resistant phenotype of Line 4-T females provided two key findings in support of the chosen congenic mapping strategy to resolve *Gct1*: (1) confirmation that the transferred genomic segment from the CAST strain encompassed the *Gct1* locus, such that the *Gct1* ^*SW*^ tumor susceptibility alleles were replaced by *Gct1* ^*CA*^ resistance alleles, and (2) confirmation that *Gct1* ^*SW*^ is the driver for the ovarian GC tumor susceptibility phenotype, despite the accumulated genetic evidence for multiple genetic modifiers of susceptibility on Chr X and several autosomes.

**Table 1:** Granulosa cell tumor incidence in SWR and Line 4-T female mice treated with testosterone or DHEA at puberty

Strain	Testosterone	DHEA
SWR	21/121 (17 %)^a^	9/51 (18 %)
Line 4-T	0/50 (0 %)	0/52 (0 %)

^a^0.1 % testosterone diet supplement (Beamer et al. 1993)

**Fig. 1:**
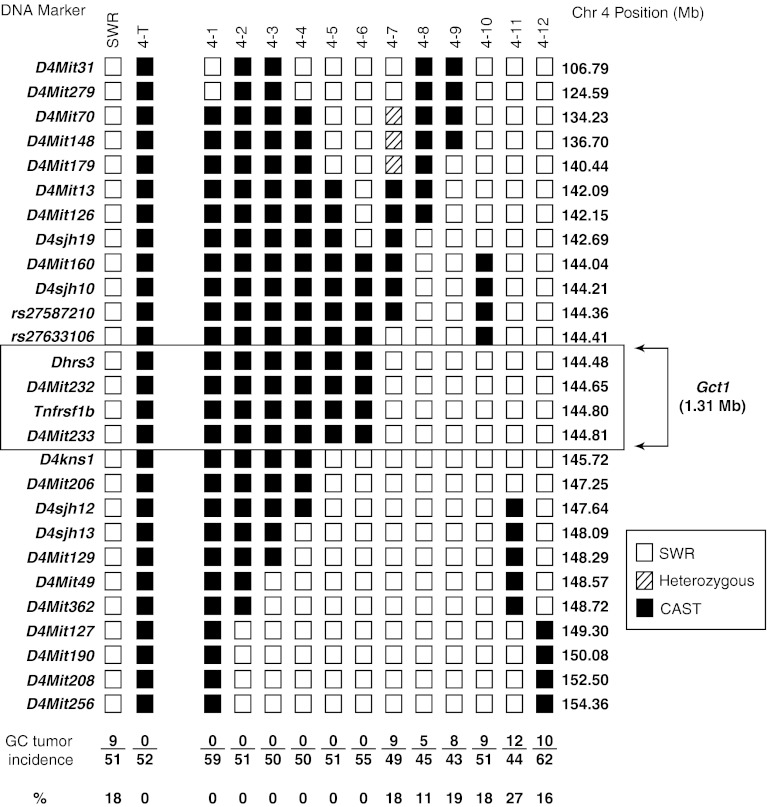
Haplotypes and GC tumor susceptibility in SWR.SJL-X.CAST founder and congenic subline females. Females from the SWR.SJL-X.CAST congenic sublines 4-7 through 4-12 have significantly increased GC tumor incidence compared with individuals from line 4-T and lines 4-1 through 4-6 (*P* *<* 0.0001). Tumor-susceptible strains share common regions of SWR genetic background between markers *rs27633106* and *D4kns1*, for a minimal 1.31 × 10^6^-bp genetic interval containing *Gct1* that is defined by line 4-10 at the proximal boundary and lines 4-5 and 4-6 at the distal boundary. Line 4-7 was found to be heterozygous for SWR and CAST alleles in the region between markers *D4Mit70* and *D4Mit179* following phenotyping. However, the heterozygous region lies outside of the *Gct1* interval and does not influence tumorigenesis. *Mb* 10^6^-bp

Twelve informative SWR.SJL-X.CAST-4 recombinant subcongenic lines (Lines 4-1 through 4-12), with unique recombinations of the *Gct1* ^*SW*^ and *Gct1* ^*CA*^ alleles across the *Gct1* locus, were successfully derived from the founder congenic Line 4-T and tested for GC tumor susceptibility with subcutaneous DHEA-filled capsules. The genotypes of the individual lines across the *Gct1* interval and the phenotype results for the groups of females examined for each line are represented in Fig. 1. Females from Lines 4-7 through 4-12 were GC tumor susceptible compared to Lines 4-1 through 4-6, which are GC tumor-resistant and therefore phenocopy Line 4-T. GC tumor incidences of the individual susceptible Lines 4-7 through 4-12 (18, 11, 19, 18, 27, and 16 %, respectively) were not different from SWR females treated with the same lot of DHEA (18 %), while the lines with a resistant phenotype (0 %) were significantly different (*P* *<* 0.0001). This indicates that the Line 4-T recombinant subcongenic Lines 4-7 through 4-12 carry the *Gct1* ^*SW*^ alleles, whereas Lines 4-1 through 4-6 retain the donor *Gct1* ^*CA*^ tumor-resistance alleles. The phenotype-driven mapping process refined the *Gct1* ^*SW*^ locus to lie between *rs27633106* and *D4kns1*, a 1.31 × 10^6^-bp interval defined by Line 4-10 at the proximal boundary and Lines 4-5 and 4-6 at the distal boundary. Of note, Line 4-7 was found to be heterozygous for SWR and CAST alleles in the region between markers *D4Mit70* and *D4Mit179* following fine-genotyping, but this region did not interfere with the *Gct1* boundary determinations.

### Short list of candidate genes for the *Gct1* tumor susceptibility locus

The 1.31 × 10^6^-bp *Gct1* interval contains 16 unique annotated genetic determinants, including 10 protein-coding genes, 2 processed transcripts, 4 noncoding RNA genes, and 23 pseudogenes (Ensembl Mouse Genome Browser release 67; NCBI m37 May 2012; Flicek et al. 2012). The majority of the annotated features are located at the distal end of the *Gct1* interval, which is particularly pseudogene-rich and contains repetitive genetic elements. Table 2 summarizes the features of the 16 annotated genes in the *Gct1* interval.

**Table 2:** Identity and features of the genetic determinants within the *Gct1* interval

Gene^a^	Description	Gene ontology	Location on chr 4 (bp)	Strand	Gene type	No. unique transcripts
*Dhrs3*	Dehydrogenase/reductase (SDR family) member 3	Oxidoreductase activity	144,482,730	+	PC	8
*Vps13d*	Vacuolar protein sorting 13 D (yeast)	ND	144,562,529	−	PC	10
*Tnfrsf1b*	Tumor necrosis factor receptor superfamily, member 1b	TNF receptor	144,803,366	−	PC	2
*Tnfrsf8*	Tumor necrosis factor receptor superfamily, member 8	TNF receptor	144,857,040	−	PC	2
*Gm13227*	Predicted gene 13227	ND	145,000,148	+	NPT	2
*Gm13225*	Predicted gene 13225	ND	145,100,662	+	PC	4
*Gm13242*	Predicted gene 13242	ND	145,104,787	+	PC	3
*Gm13212*	Predicted gene 13212	ND	145,175,069	+	PC	3
*Gm17609*	Predicted gene 17609	ND	145,191,866	−	LINC	1
*Gm13236*	Predicted gene 13236	ND	145,352,832	+	LINC	1
*AL929465.1*	–	ND	145,454,975	+	NMI	1
*Gm13235*	Predicted gene 13235	ND	145,458,697	+	PC	2
*Gm13248*	Predicted gene 13248	ND	145,539,879	−	PC	2
*1700095A21Rik*	RIKEN cDNA 1700095A21Rik gene	ND	145,651,166	+	AS	1
*Gm13247*	Predicted gene 13247	ND	145,658,647	−	PC	2
*Gm17565*	Predicted gene 17565	ND	145,686,275	+	LINC	1

*AS* novel antisense, *LINC* lincRNA, *PC* protein coding, *ND* not determined, *NMI* novel miRNA, *NPT* novel processed transcript, + forward, − reverse

^a^Excluded from this list are the 23 known pseudogenes present within the *Gct1* interval

## Discussion

Granulosa cell tumors of the sex cord-stromal class can affect women at either end of the reproductive spectrum and have generally been classified as adult- or juvenile-type based on age of onset and histology. The latest classification paradigm incorporates molecular genetic strategies, with the report of a specific association for a somatic missense (p.C134W) mutation in the *FOXL2* transcription factor in adult human GC tumors (Schrader et al. 2009; Shah et al. 2009). *FOXL2* is a member of the forkhead/hepatocyte nuclear factor 3 gene family, which is involved in normal ovarian development and GC differentiation in several species (Pisarska et al. 2011). The mechanism of the p.C134W *FOXL2* somatic mutation to support adult-type GC tumorigenesis is under investigation (Benayoun et al. 2010; D’Angelo et al. 2011; L’Hôte et al. 2012), but the curious lack of association of this genetic variant with juvenile-type GC tumors of the ovary supports the likelihood for an alternate genetic etiology in juvenile cases. Identification of *Gct1* in the SWR mouse model would provide another avenue of genetic investigation for juvenile-type GC tumors in human patients that may be dependent or independent of FOXL2 activity.

Granulosa cell tumorigenesis in the SWR mouse recapitulates inherent susceptibility to early-onset GC tumors, and genetic investigations for this polygenic ovarian trait has identified *Gct1* on distal mouse Chr 4 as a fundamental locus for GC tumor initiation during the restricted window of susceptibility at the pubertal transition. This study employed a subcongenic mapping strategy to refine the *Gct1* locus using androgen supplementation to increase trait penetrance. DHEA administration facilitated genetic mapping of *Gct1* and reinforced the extent of steroid hormone influence upon the mechanism of GC tumor initiation in the mammalian ovary.

Previous mapping studies were unable to resolve the *Gct1* interval to a practical list of candidate genes, despite strong genetic evidence for the existence of a unique tumorigenic driver determinant on distal Chr 4 in the SWR strain. The congenic mapping strategy described herein has resolved *Gct1* to a 1.31 × 10^6^-bp interval, a defined candidate gene list, and a congenic strain resource for mechanistic studies related to early-onset GC tumorigenesis. The refinement of *Gct1* was aided by the empirical identification of novel SSLP- and SNP-based genotyping DNA markers that are polymorphic between SWR and CAST strains because the published DNA marker resources had been exhausted. The 1.31 × 10^6^-bp interval currently contains annotations for 16 known protein-coding genes, known and novel processed transcripts, noncoding RNA genes, and 23 annotated pseudogenes. Until *Gct1* is identified and validated, all DNA polymorphisms unique to the SWR strain within the mapped *Gct1* interval are under consideration as causative of the trait. In SWR mice, the DHEA-responsive nature of the *Gct1* locus is an opportunity to further interrogate the list of candidate genes; however, no candidate genes within the narrowed interval are currently known to be responsive to DHEA in terms of gene expression or ligand-binding activities.

Four characterized protein-coding genes within the *Gct1* interval are conserved between mouse and human based on current genome annotations: *Vps13d*, *Tnfrsf8*, *Tnfrsf1b*, and *Dhrs3*. Given our hypothesis that mouse GC tumor-susceptibility candidates will provide translational information for juvenile-onset GC tumors of the ovary in young female patients, these genes have been given priority for further investigation based on sequence and expression analysis. A comparison of fully differentiated tissue-specific expression patterns is available for these four candidates based on the Gene Expression Atlas initiative (Su et al. 2004) accessed through the BioGPS web portal (www.biogps.gnf.org; Wu et al. 2009). The four genes show evidence of expression in both the mouse and the human ovary, in agreement with our qualitative transcript assessment in the SWR mouse ovary (data not shown). Since it is our goal to identify the mechanism of GC tumorigenesis in the SWR mouse, these genes are of significant interest, given their reported biological functions and relevant expression patterns.

*Vps13d* is a complex mouse gene with multiple isoforms. It has not been well characterized but is a member of the vacuolar-protein-sorting 13 (VPS13) gene family that is conserved across several species. In yeast, VPS proteins are involved in the trafficking of membrane proteins between the trans-Golgi network and the prevacuolar compartment. Velayos-Baeza et al. (2004) predicted through *in silico* analyses that the human VPS13D protein may have two putative domains: a ubiquitin-associated domain that confers protein target specificity in the ubiquitination pathway (Hofmann and Bucher 1996), and a ricin-B-lectin domain, which is present in many carbohydrate-recognition proteins and can bind simple sugars (Hazes 1996). It is hypothesized that if these putative domains are present, VPS13D is involved in the trafficking of ubiquitin-tagged proteins and/or carbohydrates (Velayos-Baeza et al. 2004).

*Tnfrsf8* is a member of the TNF-receptor superfamily which is expressed mainly in activated cells of the immune system (Berro et al. 2004). *Tnfrsf8* signaling can have proliferative and survival or antiproliferative and apoptotic effects depending on the cellular and stimulatory context (Gruss et al. 1994; Mir et al. 2000). Serum *Tnfrsf8* levels have been found to be increased in patients with autoimmune diseases and those infected with hepatitis B, hepatitis C, Epstein-Barr, and HIV, and *Tnfrsf8* expression is upregulated in hematological malignancies, including Hodgkin’s and non-Hodgkin’s lymphomas (reviewed by Oflazoglu et al. 2009).

*Tnfrsf1b*, a member of the TNF-receptor superfamily, is a type I transmembrane receptor that binds tumor necrosis factor alpha (TNFα). Tnfrsf1b contains a short, C-terminal intracellular region that is involved in binding TNF receptor associated factor 2 (TRAF2). TRAF2 binding triggers the recruitment of cellular inhibitor of apoptosis 1 (c-IAP1) and c-IAP2, which leads to Jun N-terminal kinase and NF-κB activation (reviewed by Carpentier et al. 2004). When signaling through Tnfrsf1b, TNFα negatively regulates ovarian folliculogenesis, as *Tnfrsf1b-*null mice had increased numbers of growing follicles compared to strain controls (Greenfeld et al. 2007); however, it is not clear if this is through an alteration of follicle growth rate, reduced follicular atresia, or some other mechanism. In humans, the p.M196R TNFRSF1B variant has been associated with hyperandrogenism and polycystic ovary syndrome in women (Peral et al. 2002). An established role for *Tnfrsf1b* in the ovary and its activation of pathways resulting in proliferation and cell survival strengthen its standing as a candidate for *Gct1* and the existence of a unique allele in the SWR strain.

The *Dhrs3* gene encodes a short-chain dehydrogenase/reductase that is induced by retinoic acid and reduces all-*trans*-retinal, a storage form of vitamin A, in a process necessary for photoreception (Haeseleer et al. 1998; Cerignoli et al. 2002). *Dhrs3* is expressed in multiple differentiated tissues besides the retina, and *DHRS3* expression has been found to be upregulated in human papillary thyroid carcinomas, although it is negatively correlated with subsequent lymph node metastasis (Oler et al. 2008). Dhrs3 was found to be a downstream target of bone morphogenic protein 2, a TGFβ family member expressed in GCs of antral follicles that regulates follicle-stimulating hormone receptor and aromatase expression and the prevention of premature follicle luteinization (Bachner et al. 1998; Shi et al. 2011). *Dhrs3* is under investigation as a candidate for shared identity with *Gct1*, as we continue to examine all candidate genes in the interval using a whole-locus customized sequencing approach, gene expression analysis, and in vivo functional strategies in GC tumor-susceptible SWR mice. We anticipate that the combination of strategies will reveal unique SWR polymorphisms, the endocrine-sensitive tumorigenic mechanism, and an explanation for trait sensitivity to DHEA.

The SWR mouse model for GC tumorigenesis is a unique and realistic model for genetically complex and stochastic cancer risk and a well-described model for juvenile GC tumors in pediatric patients. Pursuit of GC tumor susceptibility genes in the mouse will provide specific candidates for further investigation in juvenile GC tumor cases to elucidate a genetic etiology for this unique ovarian tumor class. The interval for *Gct1* is orthologous to human Chr 1p36.22, a region that has been implicated in a number of disorders through genome-wide association and cytogenetic studies. Kreisel et al. (2011) identified copy number alterations at Chr 1p36.22 in a subset of diffuse large B-cell lymphomas, an aggressive form of non-Hodgkin’s lymphoma. Human Chr 1p36.22 has also been identified as a region frequently lost in human hepatocellular carcinoma (Nishimura et al. 2006) and is a susceptibility locus for hepatocellular carcinoma in patients with hepatitis B virus infection (Zhang et al. 2010). Deletions at human Chr 1p36.22 are also frequent in infiltrating ductal carcinoma of the breast (Hawthorn et al. 2010) and premenopausal breast cancers (Varma et al. 2005). Loss of heterozygosity at the Chr 1p36.2 region is associated with neuroblastoma (Mora et al. 2000), and multiple putative tumor suppressor genes within the same region have been identified (Krona et al. 2004; Liu et al. 2011). Despite these associations, Chr 1p36.22 has not been linked with adult- or juvenile-type GC tumor susceptibility, although the rarity of cases in the human population has precluded genetic linkage studies. The identification of *Gct1* in the mouse model will permit cross-examination of the role for this ovarian GC tumor susceptibility allele in other malignancies genetically associated with Chr 1p36.22.

## Electronic supplementary material

Below is the link to the electronic supplementary material.

Supplementary material 1 (DOCX 15 kb)
