# Supplementary material for: Fine map of the Gct1 spontaneous ovarian granulosa cell tumor locus
Source: Mamm Genome. 2012 Nov 18;24(1):63–71. doi: 10.1007/s00335-012-9439-6 (PMC3560948; doi:10.1007/s00335-012-9439-6)
Supplement: Supplementary file 1 — Supplementary material 1 (DOCX 15 kb) [file 335_2012_9439_MOESM1_ESM.docx]

| Name | Forward Primer | Reverse Primer | Amplicon Size (bp) |
| --- | --- | --- | --- |
| *rs27597210* | 5'-GAAATACCAGGGGTGTGCAT | 5'-TAGGCATTGTGAGCCAGTTG | 558 |
| *rs27633106* | 5'-CAGTGGCCAGTTTTGGTTCT | 5'-CAGAGGCCAGGACTTTATGC | 336 |
| *Dhrs3* | 5'-ACATCTGGTTGTGGGAGACGGAAA | 5'-AGCCAGAGATGCTTAGGTCTGTGT | 322 |
| *Tnfrsf1b* | 5'-CACAGAGGCCCTTCAGGTTA | 5'-TAGGCTCCTCTGCCAAGTTC | 709 |
| *D4kns1* | 5'-TTTACAGAGAGAAACCCGGGCACT | 5'-ACACCCAAGTTGCGCAAGAATCTG | 376 |

Supplementary Data

Table S1: SNP-based genotyping primer sequences
